# Stunting, wasting and associated factors among children aged 6–24 months in Dabat health and demographic surveillance system site: A community based cross-sectional study in Ethiopia

**DOI:** 10.1186/s12887-017-0848-2

**Published:** 2017-04-04

**Authors:** Terefe Derso, Amare Tariku, Gashaw Andargie Biks, Molla Mesele Wassie

**Affiliations:** 1 Department of Human Nutrition, Institute of Public Health, College of Medicine and Health Sciences, University of Gondar, Gondar, Ethiopia; 2 Department of Health Service Management and Heath Economics, Institute of Public Health, College of Medicine and Health Sciences, University of Gondar, Gondar, Ethiopia

**Keywords:** Stunting, Wasting, Dabat health and demographic surveillance system, Ethiopia

## Abstract

**Background:**

Though there is a marked decline in burden of undernutrition, about 44 and 10% of children under five are stunted and wasted, respectively in Ethiopia. The highest prevalence of wasting occurs in young children (6–23 months), however literature are limited in these population groups. Therefore, this study aimed to assess stunting, wasting and associated factors among children aged 6–24 months in Dabat Health and Demographic Surveillance System (HDSS) site, northwest Ethiopia.

**Methods:**

A community based cross-sectional study was conducted in Dabat HDSS site from May 01 to June 29, 2015. A total of 587 mother-child pairs were included in the study. A multivariate logistic regression analysis was carried out to identify factors associated with stunting and wasting, separately.

**Results:**

The prevalence of stunting and wasting among children aged 6–24 months were 58.1 and 17.0%, respectively. Poor wealth status [Adjusted Odds Ratio (AOR) = 2.20; 95% Confidence Interval (CI): 1.42, 3.40], unavailability of latrine [AOR = 1.76; 95% CI: 1.17, 2.66], child age: 12–24 months [AOR = 3.24; 95% CI: 2.24, 4.69], not receiving maternal postnatal vitamin-A supplementation [AOR = 1.54; 95%: 1.02, 2.33] and source of family food: own food production [AOR = 1.71; 95% CI: 1.14, 2.57] were significantly associated with higher odds of stunting. However, only history of diarrheal morbidity was significantly associated with wasting [AOR = 2.06; 95% CI: 1.29, 3.30].

**Conclusions:**

In this community, the magnitude of stunting and wasting exists as a severe public health concern. Therefore, improving socio-economic status, latrine and maternal postnatal vitamin-supplementation coverage are essential to mitigate the high burden of stunting. Besides, reducing the childhood diarrheal morbidity as well as strengthening early diagnosis and management of the problem are crucial to curve the high prevalence of wasting.

## Background

A marked decline has been made over the past few decades in the burden of child undernutrition in developing countries, including Ethiopia [1–3]. Regardless of this success, children aged under 5 years are still suffering from undernutrition in developing countries. Globally, 178 and 52 million children are stunted and wasted, respectively [4], 99 % of this burden is found in Sub-Saharan Africa and South Asian countries [5].

Annually, about 10.5 million child deaths are related to undernutrition, in which 98% of these mortalities are reported in developing countries [6]. Stunting is associated with poor physical and cognitive development as well as low school performance in children and adolescents [7, 8]. It is also found to hinder economic productivity of adults and Gross Domestic Product (GDP) of the nation at large [9]. The risk of mortality is higher among stunted children [10], even it is aggravated when stunting and wasting happened together [11]. Due to the intergenerational impact of chronic malnutrition, childhood stunting is correlated with the future maternal undernutrition and higher risk of death [8, 12]. Premature delivery and giving birth of underweight baby are commonly reported among undernourished mothers [13, 14].

Because of their rapid growth and increased vulnerability to infectious disease, children less than 2 years of age are considered as the most at risk groups for undernutrition. As a result, the World Health Organization (WHO) designed and supports the implementation of Infant and Young Child Feeding (IYCF) Strategies [15, 16], However poor feeding practice and heath status continues as a strong determinants undernutrition [17, 18].

Accordingly, pre-lacteal feeding [19], non-exclusive breastfeeding [20], low meal frequency and dietary diversity [21–23], and not taking prenatal iron supplementation [22] are correlated with stunting among children under-5 years. Furthermore, socio-demographic and environmental factors, such as maternal illiteracy [23], young age (0–23 months) [23], occupational status (being farmer and merchant) [20, 23], large families [20, 24], poor household wealth status [25] and sanitary practice [26], unavailability of latrine [27] and use of unprotected source of drinking water [28] are identified as the determinants of stunting.

Different studies also elicited the predictors of wasting, according to which sub-optimal duration of exclusive breastfeeding [29, 30], poor wealth status [31] and maternal education [32], large families [33], diarrheal and respiratory tract morbidities [34, 35], unavailability of latrine [36] and rural residence [37] are associated with increased odds of wasting among children under-5 years.

In Ethiopia, about 44 and 10% of children under-5 years are stunted and wasted, respectively [38] and the highest burden of wasting occurs in children aged 6–23 months. More than half (53%) of child mortality [39] and 16.5% loss of the Gross Domestic Product [40] are associated with undernutrition. It was proved that, one-third of stunting and one-fourth of child deaths could be prevented by implementing basic nutrition programs [41]. Cognizant of this fact, Ethiopia is found implementing different nutritional interventions to mitigate the high burden of undernutrition [42, 43], despite significant changes have not been attained [38].

Therefore, regular monitoring of the burden and determinants of undernutrition among young children (6–24 months) is important to show the severity (public health importance) of the problem and evaluate the in-place nutritional interventions. Despite their increased and unique vulnerability, little known about the magnitude of undernutrition in this population and majority of the former local studies focused on investigating the problem among under five children [3, 17, 24, 32, 34, 36, 38]. Therefore, this study aimed to assess stunting, wasting and associated factors among children aged 6–24 months in Dabat Health and Demographic Surveillance System (HDSS) site, northwest Ethiopia.

## Methods

### Study setting and design

A community-based cross-sectional study was conducted from May 01 to June 29, 2015 in Dabat HDSS site. The HDSS site is located in Dabat District, northwest Ethiopia. The district has an estimated population size of 145,458 living in 26 rural and 4 urban kebeles *(*smallest administration unit in Ethiopia*)*. The livelihood of the residents by and large depends on subsistence farming. The HDSS site covers thirteen kebeles (four urban and nine rural kebeles) selected by considering different ecological zones (high land, middle land, and low land). A total of 67,385 people are living in these kebeles. The Dabat HDSS site has been running since November 1996, and collects information on vital events like birth, death, migration, and pregnancy registrations and its outcome on quarterly bases.

### Sample size and sampling procedure

Initially, the study was aimed to assess the nutritional status and feeding practice of children aged 6–59 months in Dabat HDSS site. Of the total kebeles (thirteen) under surveillance, eight kebeles were selected using lottery method. Accordingly, all mothers with children aged 6–59 months who lived in the selected kebeles for at least 6 months were included in the original survey. For households with multiple children fulfilling the inclusion criteria, a child was selected using lottery method.

As part of the original survey, this particular study utilized the data extracted from the original survey database. Accordingly, only mothers with children aged 6–24 months were considered to investigate the magnitude of stunting and wasting in these young children. To this effect, sample size was calculated using Epi-info version 3.7 by considering the following assumptions: 47% as the prevalence of stunting among children aged 6–24 months [3], 95% level of confidence and 5% margin of error. A design effect of 1.5 and 2.5% non-response rate were also anticipated which gives a final sample size of 588.

### Data collection tools and procedure

Data from the mothers or caregivers of the children were collected through home to home visits using structured, pretested and interviewer-administered questionnaire. The questionnaire was designed to capture socio-demographic and economic characteristics, feeding pattern, health and environmental related information. To maintain consistency, the questionnaire was first translated from English to Amharic (the native language of the study area) and was retranslated back to English. The questionnaire was pre-tested on 5% of the total sample out of the study area; accordingly the acceptability and applicability of the procedures and tools were evaluated. Fourteen data collectors and three field supervisors working in the HDSS site were recruited for the study. Training regarding anthropometric measurements and techniques of interview was given to data collectors and supervisors.

Child weight was measured to the nearest 0.1 kg by the seca beam balance (German, Serial No. 5755086138219) with graduation of 0.1 kg and a measuring range of up to 25 kg. Weight was taken with light clothing and no shoes. Instrument calibration was done before weighing each child. Furthermore, the weighing scale was checked daily against the standard weight for accuracy. The length of a child (aged 6–23 months) was measured using a horizontal wooden length board in recumbent position, and read to the nearest 0.1 cm. To check edema, normal thumb pressure was applied on both feet for 3 s. The data collectors checked whether a shallow print remained on both feet or not when the thumb was lifted.

Early initiation of breastfeeding was defined as putting the newborn to the breast within 1 h of birth. Therefore, the study participants were ask as “how long after birth did you first put [name] to the breast even if your breast milk did not arrive yet?”. If the mother responded as within 1 h of delivery, it was considered as early initiation of breastfeeding, otherwise it was defined as late initiation of breastfeeding. Similarly, initiation of complementary feeding was defined as early initiation, timely initiation and lately initiation if the mother initiated complementary feeding to the index child before sixth month, at sixth month and after sixth months of the child’s age, respectively.

A standardized seven food group containing Dietary Diversity Score (DDS) was used to qualitatively assess the dietary intake of children [44]. A 24 h dietary recall method was used, accordingly the mothers were asked to report the food items consumed by the child in the previous 24-h preceding the data of survey. By considering four food groups as the minimum acceptable dietary diversity, a child with a DDS of less than four was classified as poor dietary diversity; otherwise it was deemed to have good dietary diversity.

The household wealth index was computed using a composite indicator for urban and rural residents by considering properties such as, selected household assets and size of agricultural land. Using Principal Component Analysis (PCA), the factor scores were summed and ranked into poor, medium and rich. Data were checked for completeness and its quality, on daily basis, by the field supervisors.

### Data analysis

Data were edited, coded and entered into the EPI-info version 3.5.3 statistical software, and exported to SPSS version 20 for analysis. Nutrition related data (sex, age, height, weight and edema status) were analyzed using the WHO Anthro plus software. The Z-scores of indices, Weight-for-Height Z-score (WHZ) and Height-for-Age Z-score (HAZ) were calculated and compared using the WHO Multicenter Growth Reference Standard. A child whose HAZ less than −2 Standard Deviation (SD) from the reference population was defined as stunted, while a child with WHZ less than −2 SD from the reference population was classified as wasted [45].

Descriptive statistics, including frequencies and proportions were used to summarize the variables. Bivariate analysis was done individually for all independent variables with stunting and wasting. Variables with a *p*-values of <0.2 (child age and dietary diversity score, maternal education, occupation and postnatal vitamin-A supplementation, fathers education, household wealth status, availability of latrine, source of drinking water and main source of family food) in the bivariate analysis were entered to a multivariate logistic regression analysis to identify the independent determinants of stunting. Regarding the determinants of wasting, variables with a *P*-value of <0.2 (history of diarrheal morbidity and fever, maternal education and employment status) in the bivariate analysis were fitted to multivariate analysis. Both Crude Odds Ratio (COR) and the Adjusted Odds Ratio (AOR) with a corresponding 95% Confidence Interval (CI) were computed to show the strength of the association. In the multivariate logistic regression analysis, variables with a *p*-value of <0.05 were considered as statistically significant. Hosmer and Lemeshow goodness of fit test was checked and it was 0.77 indicating the model well fits the data.

## Results

A total of 587 mother-child pairs were included for analysis, and the mean age (±Standard Deviation, SD) of children was 15.0 (±5.7) months. Most of the mothers had no formal education (71%), were housewives (60.6%) and currently married (90.1%). Nearly three-fourth (70.4) of the households accessed food for consumption mainly from their farm while one-quarter (26.2%) through purchasing from market (Table 1).

**Table 1:** Socio-demographic and economic characteristics of children (6–24 months) and their parents, Dabat District, Northwest Ethiopia, 2015

Characteristics	Frequency	Percent
Child age in months		
6–11	196	33.4
12–24	391	66.6
Mean (±SD) age of children	(15.0 ± 5.7)	
Head of the household		
Female	18	3.1
Male	569	96.9
Mothers age		
15–34 years	371	63.2
35–50 years	216	36.8
Marital status		
Currently unmarried	58	9.9
Currently married	529	90.1
Household size		
≤ 4	224	38.2
5–7	280	47.7
8–10	83	14.1
Maternal education		
No formal education	417	71.0
Primary education	76	12.9
Secondary education	94	16.0
Maternal employment status		
Housewife	356	60.6
Farmer	143	24.4
Others^a^	88	15.0
Father education		
No formal education	389	66.3
Formal education	198	33.7
Main source of family food		
Own production	413	70.4
Purchasing	154	26.2
Others^b^	20	3.4
Wealth status		
Poor	210	35.8
Medium	198	33.7
Rich	179	30.5

^a^ Students, unemployed, servant, own business

^b^ Food donation from government and families

About half of the mothers (52.5%) took iron-folate supplements during pregnancy, but only few (1.7%) of them ate extra meal compared to their pre-pregnancy dietary habit. Though almost all of children (99.5%) were breastfed at one time in their life, only 61.5% continued to exclusively breastfed for 6 months. Only 4.3% of children consumed complementary food composed of a minimum dietary diversity. However, above three-fourth (78.9%) of children took vitamin-A supplementation in the previous 12 months prior to the data of survey while only 24.7% mothers were given the postnatal vitamin-A supplementation. One-fourth (24.4%) of the children had diarrheal morbidity in the past 2 weeks prior to the date of survey (Table 2). Despite two-third of households used unsafe source of water for consumption, only 2.4% mothers were found to treat water on regular basis (always) before consumption. Latrine was also available in 28.1% of the households (Table 3).

**Table 2:** Feeding practice, micronutrient supplementation and morbidity related characteristics of study participants, Dabat District, Northwest Ethiopia, 2015

Characteristics	Frequency	Percent
Feeding during pregnancy		
Less than before/usual	356	60.6
As usual	221	37.6
Greater than usual/extra foods	10	1.7
Prenatal iron-folate supplementation		
Yes	308	52.5
No	279	47.5
Colostrum		
Given to the child	324	55.2
Discarded	263	44.8
Ever breastfeeding		
Yes	584	99.5
No	3	0.5
Initiation of breastfeeding		
Early initiation	333	57.0
Late initiation	251	43.0
Exclusive breastfeeding		
Yes	361	61.5
No	226	38.5
Pre-lacteal feeding		
Yes	137	23.3
No	450	76.7
Complementary feeding initiation		
Early initiation	109	18.6
Timely initiation	314	53.5
Lately initiation	164	27.9
Bottle feeding in the previous 24 h		
Yes	40	6.8
No	547	93.2
Dietary diversity score		
< 4 food groups	562	95.7
≥ 4 food groups	25	4.3
Maternal Vitamin A supplementation		
Yes	145	24.7
No	442	75.3
History of fever in the previous 2 weeks		
Yes	237	40.4
No	350	59.6
History of diarrheal disease in the previous 2 weeks		
Yes	143	24.4
No	444	75.6
Child vitamin A supplementation in the previous 12 months		
Yes	463	78.9
No	124	21.1

**Table 3:** Environmental health related characteristics of the households, Dabat District, northwest Ethiopia, 2015

Characteristics	Frequency	Percent (%)
Source of drinking water		
Safe source^a^	212	36.1
Unsafe source^b^	375	63.9
Water treatment habit		
Not at all	550	93.7
Sometimes	23	3.9
Always	14	2.4
Availability of latrine		71.9
Yes	165	28.1
No	422	71.9
Waste disposal		
Appropriate^c^	74	12.6
Inappropriate^d^	513	87.4
Hand washing habit after toilet		
Not at all	71	12.1
Sometimes	111	18.9
Always	405	69

^a^ Piped, protected well and spring water

^b^ Unprotected well, spring and surface water

^c^ Collected by municipality, buried and burned

^d^ Dumped in street/open space, compound and river

The prevalence of stunting and wasting among children aged 6–24 months were 58.1% [95% CI; 50.3, 65.9%] and 17.0% [95% CI; 11.1, 22.9%], respectively. Besides, the burden of stunting and wasting among children aged 12–24 months was 44.6 and 11.6%, respectively, while it was 13.5 and 5.4% in infants aged 6–11 months.

In the bivariate analysis, child age, maternal education, occupation and postnatal vitamin-A supplementation, fathers’ education, household wealth status, availability of latrine and main source of family food were significantly associated with stunting. However, the multivariate logistic regression analysis detected that child age, maternal postnatal vitamin-A supplementation, latrine availability, main source of family food and household wealth status were remained significantly and independently associate with stunting.

Thus, the odds of stunting were 54% [AOR = 1.54; 95%: 1.02, 2.33] higher among children whose mothers did not receive postnatal vitamin-A supplementation compared to their counterparts. The odds of stunting among children aged 12–24 months were 3.24 [AOR = 3.24; 2.24, 4.69] times higher compared to infants aged 6–11 months. The higher likelihood of stunting was found among children from households without latrine [AOR = 1.76; 95% CI: 1.17, 2.66] and with poor wealth status [AOR = 2.20; CI: 1.42, 3.40]. Finally, the odds of stunting were 71% higher among children whose families accessed food for household consumption from their own farm [AOR = 1.71; 95% CI: 1.14, 2.57] compared to those children whose families accessed food from other sources (Table 4).

**Table 4:** Factors associated with stunting among children aged 6–24 months, Dabat District, Northwest Ethiopia, 2015

Variables	Stunting		COR [95% CI]	AOR [95% CI]
Maternal education	Yes (#)	No (#)		
No formal education	254	163	2.20 (1.39,3.46)	1.56 (0.91,2.69)
Primary education	48	28	2.42 (1.30,4.50)	1.84 (0.92,3.63)
Secondary and above education	39	55	1.00	1.00
Maternal occupation				
Housewife	201	155	1.49 (0.93,2.37)	0.96 (0.52,1.78)
Farmer	99	44	2.58 (1.49,4.47)	1.41 (0.69,2.89)
Others^a^	41	47	1.00	1.00
Father education				
No formal education	238	151	1.45 (1.03,2.05)	1.09 (0.72,1.66)
Formal education	103	95	1.00	1.00
Wealth status				
Poor	143	67	2.36 (1.56,3.57)	2.20 (1.42,3.40)^*^
Medium	113	85	1.43 (0.98,2.21)	1.30 (0.83,2.02)
Rich	85	94	1.00	1.00
Main source of family food				
Own production	256	157	1.71 (1.91,2.44)	1.71 (1.14,2.57)^*^
Others^b^	85	89	1.00	1.00
Source of drinking water				
safe source	111	101	1.00	1.00
unsafe source	230	145	1.44 (1.03,2.03)	1.21 (0.79,1.84)
Availability of latrine				
Yes	77	88	1.00	1.00
No	264	158	1.19 (1.33,2.75)	1.76 (1.17,2.66)^*^
Maternal Vitamin A supplementation				
Yes	73	72	1.00	1.00
No	268	174	1.52 (1.04,2.22)	1.54 (1.02,2.33)^*^
Dietary diversity score				
< 4 food groups	330	232	1.81 (0.81,4.06)	1.17 (0.69,4.04)
≥ 4 food groups	11	14	1.00	1.00
Child age				
6–11 months	79	117	1.00	1
12–24 months	262	129	3.01 (2.12,4.29)	3.24 (2.24,4.69) ^*^

^*^ Significant at *p*-value < 0.05

^a^ Students, unemployed, servant, own business

^b^ Purchasing, and donation from government or families

Regarding the determinants of wasting, both bivariate and multivariate analyses indicated that only diarrheal morbidity remained significantly and independently associated with wasting. Accordingly, the odds of wasting were 2.06 times [AOR = 2.06; CI: 1.29, 3.30] higher among children with history of diarrheal disease compared to those who had no history of diarrheal morbidity in the past 2 weeks prior to the date survey (Table 5).

**Table 5:** Factors associated with wasting among children aged 6–24 months in Dabat District, Northwest, Ethiopia, 2015

Variables	Wasting		COR (95%CI)	AOR (95%CI)
Maternal education	Yes (#)	No (#)		
No formal education	64	353	0.80 (0.34,1.03)	0.67 (0.35,1.25)
Primary education	14	62	0.74 (0.35,1.57)	0.73 (0.33,1.60)
Secondary education and above	22	72	1.00	1.00
Maternal occupation				
Housewife	49	307	0.55 (0.32,1.05)	0.63 (0.34,1.14)
Farmer	32	111	1.05 (0.55,1.99)	1.21 (0.63,2.33)
Others	19	69	1.00	1.00
History of fever in the past 2 weeks				
Yes	49	188	1.53 (0.99,2.34)	1.07 (0.61,1.86)
No	51	299	1.00	1.00
Diarrheal morbidity in the past 2 weeks				
No	64	380	1.00	1.00
Yes	36	107	2.00 (1.26,3.16)	2.06 (1.29,3.30)^*^

^*^Significant at a *P*-value of <0.05

## Discussion

This study revealed that, the prevalence of stunting was 58.1% among children aged 6–24 months. The current burden of stunting suggests the severe public health significance of the problem [46]. Similar magnitude of stunting was also reported in Central Africa Republic (61.5%) [47] and Kenya (51%) [48]. However, the finding was higher than what was reported in Egypt (20.3%) [49]. The discrepancy might be related to variations in residence and level of income between the study participants. In contrast to the latter study, the participants of the current study were majorly rural inhabitants. Poor child feeding and caring practice and health care access is documented in the rural Ethiopia [38]. This suggests that unequal allocation of resources cause to inequalities in child health outcomes between rural and urban settlements [50]. In addition, stunting is more common in the rural areas [51]. On the other hand, the livelihood of the study participants is mainly depend on subsistence, low-yield and rain-fed farming. The output of subsistence farming is mostly for local requirement with little or no surplus for trade [52], which ultimately impairs the household food security status. Household food insecurity is associated with poor linear growth, stunting [53].

Compared to the WHO cut-off point to declare the public health importance of undernutrition, the prevalence of wasting (17.0%) existed as a critical public health problem [46]. The finding was in line with reports of other developing countries, such as Central Africa Republic (20.2%) [47] and Sri Lanka (17.1%) [54]. However, compared to this study, the lower prevalence of wasting is reported in the rural Cambodia (10%) [55]. The study in Cambodia was conducted among Food and Agriculture Organization (FAO) project beneficiaries which aimed to promote optimal IYCF practice and improve the household food security status through increasing market availability, production and diversification of food. Research findings from different low and middle income countries showed that improved agriculture is one of the key nutrition sensitive interventions to rectify maternal and child undernutrition [56–58]. However, the agricultural practice in Ethiopia, including the study area, is rain feed, based on poor technologies, and there is also poor market regulation system for food products [59, 60].

The result of multivariable analysis showed that, the likelihood of being stunted was 2.20 times higher among children from the poor families compared to those from the rich families. The finding was supported by the reports elsewhere [61, 62]. The plausible explanation might be related to the negative effect of poor wealth status on the household food access, utilization of health services, availability of improved water sources, and sanitation facilities [63].

The results of the present study also detected that, the odds of stunting were increased among children from a household without latrine. Similarly, the previous reports in Africa indicated the decreased odds of childhood stunting in households with latrine [62, 64]. In fact, unfavorable health environment caused by inadequate sanitation increases vulnerability to communicable diseases which is considered as immediate causes of undernutrition, including stunting [63].

The uncommon finding of this study was increased odds of stunting among children whose families accessed food mainly from farm (own production). This might be due to low agricultural productivity and poor complementary feeding practice in the study area. In fact, own production of food does not necessarily mean ensuring per-capita food availability and nutritional security. In Ethiopia, including the study area, complementary food is mainly prepared from cereal (Teff, barely and sorghum) based food products where the traditional staple food called ‘Injera’ (a yeast-risen flatbread made of a blend of cereals*)* is usually served with legumes or pulses. Such feeding practices increase the vulnerability of infants and children to poor energy and micronutrient intake [65].

Children whose mothers did not receive the postnatal vitamin-A supplementation were found with increased odds of stunting compared to their counterparts. Improving vitamin-A status of children is one of the proven child survival strategies, specially it is found to significantly reduce risk of morbidity and mortality from infectious diseases [66, 67]. Frequent episodes of infectious disease, such as diarrhea and respiratory tract infections are strongly associated with higher risk of stunting [68]. In Ethiopia, most of the pregnant mothers are suffering from vitamin-A deficiency [69]. As a result, postnatal period is a window of opportunity to improve mothers vitamin-A status thereby increasing the retinol level of breast milk. By doing so, the breastfed infants will get adequate amount of vitamin-A which further helps to reduce the risk of infectious disease episodes through boosting their immunity.

Likewise, the odds of stunting were higher among children aged 12–24 months compared to those aged 6–11 months. This finding was in agreement with the report from Central Africa Republic, in which poor growth of children is correlated with old age of children [47]. Studies of other developing countries also claimed that stunting is less common in early infancy as they are on breastfeeding [70], however, because of inappropriate complementary feeding practice and higher nutritional demand, the risk of impaired linear growth increases as the child’s age advances [71].

Finally, this study reported that, children with history of diarrheal morbidity in the previous 2 weeks preceding the date survey were found with higher odds of developing wasting. Similar findings were also reported by the previous local studies [24, 34]. Obviously, diarrhea is associated with malabsorption of nutrients, significant nutrient and fluids loss and reduced appetite [72]. Delayed treatment due to mothers poor health seeking behavior and inappropriate home based management of diarrhea, such as fluid restriction and decreasing or stopping food intake are the commonly practiced in most of the mothers in Ethiopia, which in turn increases the vulnerability the child to develop wasting [73, 74].

This is one of the few studies revealing the magnitude of stunting and wasting in the most vulnerable population groups, children aged 6–24 months. Nevertheless, it is not free from some of limitations. Firstly, though intensive training, regular field supervision and pre-test were done, the study was not free from measurement and recall bias while conducting anthropometric and child feeding practice assessments, respectively.

## Conclusions

The prevalence of stunting and wasting are high in Dabat HDSS site which indicates that undernutrition is the severe public health concern among children. In addition, household wealth status, latrine availability, maternal postnatal vitamin-A supplementation, child age and main source of family food were significantly associated with stunting. But, only diarrheal morbidity was identified as the key determinant of wasting. Therefore, improving socio-economic status, latrine and maternal postnatal vitamin-supplementation coverage are essential to mitigate the high burden of stunting. It is also crucial to strengthen the implementation of the current measures focusing on reducing the occurrence of childhood diarrheal morbidity as well as early diagnosis and management of the problem.

## Abbreviations

PCA: Principal Component Analysis
GDP: Gross domestic production
WHO: World Health Organization
AOR: Adjusted Odds Ratio
COR: Crude Odds Ratio
CI: Confidence Interval
IYCF: Infant and young child feeding
DDS: Dietary Diversity Score
SD: Standard Deviation
HDSS: Health and Demographic Surveillance System

## References

[1] de Onis M, Blössner M (1997). WHO global database on child growth and malnutrition.

[2] Smith LC, Haddad LJ. Overcoming child malnutrition in developing countries: past achievements and future choices. Intl Food Policy Res Inst. 2000;30:1–6.

[3] Central Statistical Authority [Ethiopia] and ORC Macro (2014). Mini Ethiopia demographic and health survey 2014. Addis Ababa. Maryland: Ethiopia and Calverton.

[4] Black RE, Victora CG, Walker SP, Bhutta ZA, Christian P, De Onis M, Ezzati M, Grantham-McGregor S, Katz J, Martorell R (2013). Maternal and child undernutrition and overweight in low-income and middle-income countries. Lancet.

[5] De Onis M, Brown D, Blossner M, Borghi E (2012). Levels and trends in child malnutrition. UNICEF-WHO-the World Bank joint child malnutrition estimates.

[6] UNICEF. The state of the world's children 2007: women and children: the double dividend of gender equality. New York: UNICEF; 2006.

[7] Pollitt E, Gorman KS, Engle PL, Martorell R, Rivera J, Wachs TD, Scrimshaw NS. Early supplementary feeding and cognition: effects over two decades. Monogr Soc Res Child Dev. 1993;58(7):i-118. 8272081

[8] Moock PR, Leslie J (1986). Childhood malnutrition and schooling in the Terai region of Nepal. J Dev Econ.

[9] Grantham-McGregor S, Cheung YB, Cueto S, Glewwe P, Richter L, Strupp B, Group ICDS (2007). Developmental potential in the first 5 years for children in developing countries. Lancet.

[10] Olofin I, McDonald CM, Ezzati M, Flaxman S, Black RE, Fawzi WW, Caulfield LE, Danaei G, Study NIM (2013). Associations of suboptimal growth with all-cause and cause-specific mortality in children under five years: a pooled analysis of ten prospective studies. PLoS One.

[11] McDonald CM, Olofin I, Flaxman S, Fawzi WW, Spiegelman D, Caulfield LE, Black RE, Ezzati M, Danaei G (2013). The effect of multiple anthropometric deficits on child mortality: meta-analysis of individual data in 10 prospective studies from developing countries. Am J Clin Nutr.

[12] Black RE, Allen LH, Bhutta ZA, Caulfield LE, De Onis M, Ezzati M, Mathers C, Rivera J, Maternal, Group CUS (2008). Maternal and child undernutrition: global and regional exposures and health consequences. Lancet.

[13] Soon BT (2012). The global action report on preterm birth: Eds. Howson CP, Kinney MV, Lawn JE. March of Dimes, PMNCH, save the children.

[14] Shekar M, Heaver R, Lee Y-K. Repositioning nutrition as central to development: A strategy for large scale action. Washington DC: World Bank Publications; 2006.

[15] World Health Organization. Global strategy for infant and young child feeding: UNICEF. Singapore: World Health Organization; 2003.

[16] WHO U, IFPRI U, Davis U (2008). FANTA: indicators for assessing infant and young child feeding practices.

[17] Getahun Z, Urga K, Genebo T, Nigatu A (2001). Review of the status of malnutrition and trends in Ethiopia. Ethiop J Health Dev.

[18] Kumar D, Goel N, Mittal PC, Misra P (2006). Influence of infant-feeding practices on nutritional status of under-five children. Indian J Pediatr.

[19] Asfaw M, Wondaferash M, Taha M, Dube L (2015). Prevalence of undernutrition and associated factors among children aged between six to fifty nine months in Bule Hora district, South Ethiopia. BMC Public Health.

[20] Disha A, Rawat R, Subandoro A, Menon P (2012). Infant and young child feeding (IYCF) practices in Ethiopia and Zambia and their association with child nutrition: analysis of demographic and health survey data. Afr J Food Agric Nutr Dev.

[21] Agho KE, Inder KJ, Bowe SJ, Jacobs J, Dibley MJ (2009). Prevalence and risk factors for stunting and severe stunting among under-fives in North Maluku province of Indonesia. BMC Pediatr.

[22] Bwalya BB, Lemba M, Mapoma CC, Mutombo N (2015). Factors associated with stunting among children aged 6-23 months in Zambian: evidence from the 2007 Zambia demographic and health survey. Int J Adv Nutr Health Sci.

[23] Agedew E, Chane T. Prevalence of stunting among children aged 6–23 months in Kemba Woreda, Southern Ethiopia: a community based cross-sectional study. Adv Public Health. 2015;2015:1–6.

[24] Tamiru MW, Tolessa BE, Abera SF. Under nutrition and associated factors among under-five age children of Kunama Ethnic Groups in Tahtay Adiyabo Woreda, Tigray Regional State, Ethiopia: Community Based Study; 2015.

[25] Tiwari R, Ausman LM, Agho KE (2014). Determinants of stunting and severe stunting among under-fives: evidence from the 2011 Nepal demographic and health survey. BMC Pediatr.

[26] Vitolo MR, Gama CM, Bortolini GA, Campagnolo PD, MDL D (2008). Some risk factors associated with overweight, stunting and wasting among children under 5 years old. J Pediatr.

[27] Mostafa Kamal SM (2011). Socio-economic determinants of severe and moderate stunting among under-five children of rural Bangladesh.

[28] Chirande L, Charwe D, Mbwana H, Victor R, Kimboka S, Issaka AI, Baines SK, Dibley MJ, Agho KE (2015). Determinants of stunting and severe stunting among under-fives in Tanzania: evidence from the 2010 cross-sectional household survey. BMC Pediatr.

[29] Onsa ZO, Ahmed NMK (2014). Impact of exclusive breast feeding on the growth of Sudanese children (0-24 months). Pak J Nutr.

[30] Sreedhara M, Banapurmath C (2013). A study of nutritional status of infants in relation to their complementary feeding practices. Nature.

[31] Engle PL, Menon P, Haddad L (1999). Care and nutrition: concepts and measurement. World Dev.

[32] Bantamen G, Belaynew W, Dube J (2014). Assessment of factors associated with malnutrition among under five years age children at Machakel Woreda, Northwest Ethiopia: a case control study. J Nutr Food Sci.

[33] Ayana AB, Hailemariam TW, Melke AS (2015). Determinants of acute malnutrition among children aged 6–59 months in public hospitals, Oromia region, West Ethiopia: a case–control study. BMC Nutr.

[34] Amsalu S, Tigabu Z (2008). Risk factors for severe acute malnutrition in children under the age of five: a case control study. Ethiop J Health Dev.

[35] Gebretsadik A, Worku A, Berhane Y, Morries L, Cassano P, Henderson T, Stalker C, Elander J. Factors associated with acute respiratory infection in children under the age of 5 years: evidence from the 2011 Ethiopia demographic and health survey [corrigendum]. Neuropsychiatr Dis Treat. 2015(11):2159–75.

[36] Tariku B, Mulugeta A, Tsadik M, Azene G (2014). Prevalence and risk factors of child malnutrition in community based nutrition program implementing and Nonimplementing districts from south East Amhara, Ethiopia. Open Access Libr J.

[37] Fotso J-C (2007). Urban–rural differentials in child malnutrition: trends and socioeconomic correlates in sub-Saharan Africa. Health Place.

[38] Central Statistical Authority [Ethiopia] and ORC Macro (2011). Ethiopia demographic and health survey 2011. Addis Ababa. Maryland: Ethiopia and Calverton.

[39] Federal Ministry of Health, Family Health Department Ethiopia (2004). National strategy for infant and young child feeding.

[40] UNICEF. The cost of hunger in Ethiopia. The social and economic impact of child undernourishment in Ethiopia summary report. Addis Ababa: UNICEF; 2014.

[41] Bhutta ZA, Ahmed T, Black RE, Cousens S, Dewey K, Giugliani E, Haider BA, Kirkwood B, Morris SS, Sachdev H (2008). What works? Interventions for maternal and child undernutrition and survival. Lancet.

[42] Federal Democratic and Republic of Ethiopia: National Nutrition Program: 2015. https://www.usaid.gov/ethiopia/press-releases/ethiopia-launches-new-multi-sector-national-nutrition-program.

[43] Federal Ministry of Health (2004). Ethiopian National Strategy on infant and young child feeding.

[44] World Health Organization (2010). Indicators for assessing infant and young child feeding practices part 3: country profiles.

[45] World Health Organization, UNICEF (2009). WHO child growth standards and the identification of severe acute malnutrition in infants and children: joint statement by the World Health Organization and the United Nations Children's fund.

[46] WHO (2010). Nutrition landscape information system (NLIS) country profile indicators: interpretation guide.

[47] Wanga Y, Tokunaga M, Ikuta S. Factors associated with nutritional status in children aged 6–24 months in Central African Republic-An anthropometric study at health centers in Bangui. J Int Health. 2009;24(4):289–98.

[48] Adeladza A. The influence of socio-economic and nutritional characteristics on child growth in Kwale District of Kenya. Afr J Food Agr Nutr Dev. 2009;9(7):570–90.

[49] Seedhom AE, Mohamed ES, Mahfouz EM (2014). Determinants of stunting among preschool children, Minia, Egypt. Int Public Health Forum.

[50] Siddiqi A, Hertzman E, Irwin LG, Hertzman C (2012). Early child development: a powerful equalizer. Improving equity in health by addressing social determinants.

[51] De Onis M, Blössner M, Borghi E (2012). Prevalence and trends of stunting among pre-school children, 1990–2020. Public Health Nutr.

[52] Ethiopia trade and transformation: Diagnostic trade integration study. Addis Ababa; 2004.

[53] Ali Naser I, Jalil R, Muda W, Manan W, Nik W, Suriati W, Mohd Shariff Z, Abdullah MR (2014). Association between household food insecurity and nutritional outcomes among children in northeastern of peninsular Malaysia. Nutr Res Pract.

[54] Ubeysekara NH, Jayathissa R, Wijesinghe CJ (2015). Nutritional status and associated feeding practices among children aged 6-24 months in a selected community in Sri Lanka: a cross sectional study. Eur J Prev Med.

[55] Reinbott A, Kuchenbecker J, Herrmann J, Jordan I, Muehlhoff E, Kevanna O, Krawinkel M (2015). A child feeding index is superior to WHO IYCF indicators in explaining length-for-age Z-scores of young children in rural Cambodia. Paediatr Int Child Health.

[56] Ruel MT, Alderman H, Maternal, Group CNS (2013). Nutrition-sensitive interventions and programmes: how can they help to accelerate progress in improving maternal and child nutrition?. Lancet.

[57] Gillespie S, Haddad L, Mannar V, Menon P, Nisbett N (2013). The politics of reducing malnutrition: building commitment and accelerating progress. Lancet.

[58] Bhutta ZA, Das JK, Rizvi A, Gaffey MF, Walker N (2013). Evidence-based interventions for improvement of maternal and child nutrition: what can be done and at what cost?. Lancet.

[59] Von Braun J, Olofinbiyi T (2007). Famine and food insecurity in Ethiopia “FOOD POLICY FOR DEVELOPING COUNTRIES : THE ROLE OF GOVERNMENT IN THE GLOBAL FOOD SYSTEM”.

[60] Endalew B, Muche M, Tadesse S (2015). Assessmen of food security situation in Ethiopia: a review Asian Journal of Agricalture Research.

[61] Victora CG, Vaughan JP, Kirkwood BR, Martines JC, Barcelos LB (1986). Risk factors for malnutrition in Brazilian children: the role of social and environmental variables. Bull World Health Organ.

[62] Getaneh T, Assefa A, Tadesse Z (1998). Protein-energy malnutrition in urban children: prevalence and determinants. Ethiop Med J.

[63] UNICEF (1990). Strategies of improving nutrition of children and women in developing countries.

[64] Sommerfelt A, KStewart M (1994). Children’s nutritional status. Demographic and health surveys comparative studies no. 12. Calverton, MD: Macro International. Inc, Zimbabwe.

[65] Mekonnen A, Jones N, Tefera B (2005). Tackling child malnutrition in Ethiopia: do the sustainable development poverty reduction programme's underlying policy assumptions reflect local realities?.

[66] Vitamin A Global Initiative. A strategy for acceleration of progress in combating Vitamin A deficiency. Consensus of an Informal Technical Consultation. New York: UNICEF; 1997.

[67] Ezzati M, Lopez AD, Rodgers A, Murray CJ. Comparative quantification of health risks. Global and regional burden of disease attributable to selected major risk factors Geneva: World Health Organization; 2004. pp. 1987–97.

[68] Pinstrup Andersen P, Burger S, Habicht JP, Peterson K. Protein–energy malnutrition. In: Jamison DT, Mosley WH, Measham AR, Bobadilla JL, editors. Disease control priorities in developing countries. 2nd ed. Oxford (UK): Oxford University Press; 1993. pp. 391-420.

[69] Gebreselassie SG, Gase FE, Deressa MU (2013). Prevalence and correlates of prenatal vitamin a deficiency in rural Sidama, southern Ethiopia. J Health Popul Nutr.

[70] Thiombiano-Coulibaly N, Rocquelin G, Eymard-Duvernay S (2004). Effects of early extra fluid and food intake on breast milk consumption and infant nutritional status at 5 months of age in an urban and a rural area of Burkina Faso. Eur J Clin Nutr.

[71] Ricci JA, Becker S (1996). Risk factors for wasting and stunting among children in metro Cebu, 72. 65.Philippines. Am J Clin Nutr.

[72] Ahmed T, Ali M, Ullah MM, Choudhury IA, Haque ME, Salam MA, Rabbani GH, Suskind RM, Fuchs GJ (1999). Mortality in severely malnourished children with diarrhoea and use of a standardised management protocol. Lancet.

[73] Tessema F, Asefa M, Ayele F (2002). Mothers’ health services utilization and health care seeking behaviour during infant rearing: a longitudinal community based study, south west Ethiopia. Ethiop J Health Dev.

[74] Olango P, Aboud F (1990). Determinants of mothers’ treatment of diarrhea in rural Ethiopia. Soc Sci Med.

